# Selected Cytokines and Metalloproteinases in Inflammatory Bowel Disease

**DOI:** 10.3390/ijms25010202

**Published:** 2023-12-22

**Authors:** Barbara Sosna, David Aebisher, Angelika Myśliwiec, Klaudia Dynarowicz, Dorota Bartusik-Aebisher, Piotr Oleś, Grzegorz Cieślar, Aleksandra Kawczyk-Krupka

**Affiliations:** 1 Department of Internal Medicine, Angiology and Physical Medicine, Center for Laser Diagnostics and Therapy, Medical University of Silesia in Katowice, Batorego 15 Street, 41-902 Bytom, Poland; barbara.sosna@hotmail.com (B.S.); piotroles@o2.pl (P.O.); cieslar1@tlen.pl (G.C.); 2 Department of Photomedicine and Physical Chemistry, Medical College, University of Rzeszów, 35-959 Rzeszów, Poland; daebisher@ur.edu.pl; 3 Center for Innovative Research in Medical and Natural Sciences, Medical College, University of Rzeszów, 35-310 Rzeszów, Poland; amysliwiec@ur.edu.pl (A.M.); kdynarowicz@ur.edu.pl (K.D.); 4 Department of Biochemistry and General Chemistry, Medical College, University of Rzeszów, 35-959 Rzeszów, Poland; dbartusikaebisher@ur.edu.pl

**Keywords:** inflammatory bowel disease, IBD, UC, CD, cytokines, metalloproteinases, MMPs

## Abstract

Inflammatory bowel disease (IBD) is a collective term for two diseases: ulcerative colitis (UC) and Crohn’s disease (CD). There are many factors, e.g., genetic, environmental and immunological, that increase the likelihood of these diseases. Indicators of IBDs include extracellular matrix metalloproteinases (MMPs). The aim of this review is to present data on the role of selected cytokines and metalloproteinases in IBD. In recent years, more and more transcriptomic studies are emerging. These studies are improving the characterization of the cytokine microenvironment inside inflamed tissue. It is observed that the levels of several cytokines are consistently increased in inflamed tissue in IBD, both in UC and CD. This review shows that MMPs play a major role in the pathology of inflammatory processes, cancer, and IBD. IBD-associated inflammation is associated with increased expression of MMPs and reduced ability of tissue inhibitors of metalloproteinases (TIMPs) to inhibit their action. In IBD patients in tissues that are inflamed, MMPs are produced in excess and TIMP activity is not sufficient to block MMPs. This review is based on our personal selection of the literature that was retrieved by a selective search in PubMed using the terms “Inflammatory bowel disease” and “pathogenesis of Inflammatory bowel diseases” that includes systematic reviews, meta-analyses, and clinical trials. The involvement of the immune system in the pathophysiology of IBD is reviewed in terms of the role of the cytokines and metalloproteinases involved.

## 1. Introduction

Ulcerative colitis (UC) and Crohn’s disease (CD) are categorized as inflammatory bowel diseases (IBD). Both these diseases are characterized by a chronic process, during which there are periods of exacerbation and remission. Genetic, environmental, and immunological factors have a significant impact on the pathogenesis of inflammatory bowel diseases. Crohn’s disease is an autoimmune disease of the intestines. The improper functioning of the immune system is probably due to the body’s inappropriate reaction to micro-organisms inhabiting the digestive tract. Autoimmune diseases develop under the influence of environmental factors in genetically predisposed people. The main cause is impaired immune tolerance—the body incorrectly recognizes its own antigens. Autoimmune diseases may affect single organs or entire systems [1]. Overall, it is estimated that IBDs have been diagnosed in 0.3% of the European population, which corresponds to a total of 3 million people, and it is estimated that the incidence will increase by almost a quarter between 2017 and 2025 [1], making them civilization diseases. The increase in illness across Europe is occurring mainly in the newly industrialized countries of Eastern Europe, which has been linked to changing environmental factors [2,3,4]. Currently, treatment decisions are based on clinical symptoms, laboratory indicators, gastrointestinal imaging and endoscopic studies, but the results of the performed tests do not always correlate with the stage of the disease, resulting in frequent failure to achieve remission after treatment. Therefore, the search for new biomarkers of IBD activity capable of assessing the severity of the underlying disease in a minimally invasive manner in everyday practice, is currently of interest to researchers around the world. This study presents the characteristics of selected cytokines and metalloproteinases as well as their importance in IBD.

## 2. Characteristics of IBD

### 2.1. Ulcerative Colitis

Ulcerative colitis is characterized by the fact that it is a recurrent IBD. Its characteristics are mucosal inflammation. It starts distally but, consequently, can reach the proximal area, that is, reaching the colon [5].

**Figure 1:**
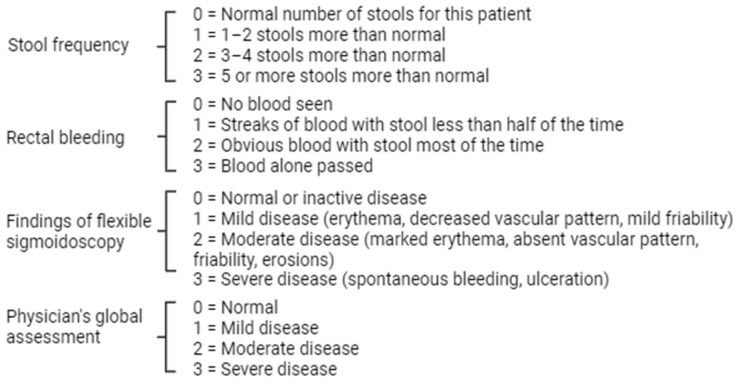
Scoring system for assessment of UC activity [1,2,3,4,5,6,7].

The extent of disease activity is a cross-sectional of events at a specific time of inflammation, although disease severity may include other types of factors (such as longitudinal—previous biological failure, maximum history of disease severity, and indicators of health care use such as hospitalization and disability assessment tools and historical factors) [6]. One of the most popular and widely used scales in clinical practice is the Mayo scale (Figure 1).

The Mayo scale, like other methods, includes several variables characterized by high inter-observer variability (i.e., mucosal fragility). In 2012, the Endoscopic Ulcerative Colitis Severity Index (UCEIS) was developed [7]. According to this index, only 3 descriptors, namely, the vascular system, bleeding, and erosions and ulcers, can suffice to create a model that accounts for 90% of the overall endoscopic severity score, which is closely related to UC (Table 1) [1,2,3,4].

Disease severity is measured by assessing parameters of a clinical and biochemical nature, as is presented in the modified Truelove and Witts criteria (Table 2) [8,9]. In the endoscopic field, UCEIS is the only validated system for assessing disease severity; however, the Mayo scale is widely used in clinical practice because of its simplicity of use [8,10].

At least 30 histologic scoring systems have been developed, but they have some form of validation [11]. Validated results include the Truelove and Richards index (tab above), the Gomes index [12], the Riley scale [13], the Geboes scale [14], the Harpaz/Mount Sinai index [15], the modified Riley scale [16], the Chicago/Rubin/Histologic inflammation activity scale, the modified Harpase index [17], the simplified Geboes score [18], the Nancy index [19], and the Robarts histopathology score [20].

But none of the above assessments have been fully validated. As an example of evaluating histological findings in clinical trial data, the research group reported combined histological and endoscopic findings. Mucosal healing was defined, having features: (1) neutrophil infiltration in less than 5% of crypts; (2) no destruction of the crypt; and (3) no erosion, ulceration, or granulation tissue with endoscopic improvement [20]. In addition, histologic remission is correlated with endoscopic improvement, higher rates of sustained steroid-free remission, and reduced rates of clinical recurrence and hospitalization [21,22,23,24].

### 2.2. UC Classification

**Figure 2:**
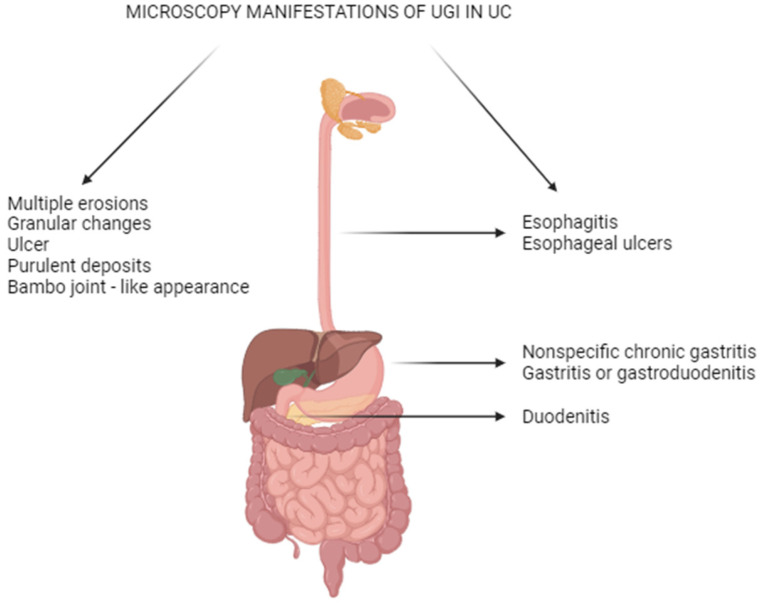
Selected endoscopic and microscopic upper gastrointestinal signs in UC.

The Montreal classification divides patients with UC based on maximal disease severity into E1 or rectal inflammation; E2 or left-sided disease; and E3 or extensive colitis [8]. The figure below (Figure 2) shows only selected endoscopic and microscopic signs in the upper gastrointestinal area in UC.

There are several tests that are used to diagnose UC. Table 3 shows the types of these tests and their brief characteristics.

### 2.3. Etiopathogenesis

**Figure 3:**
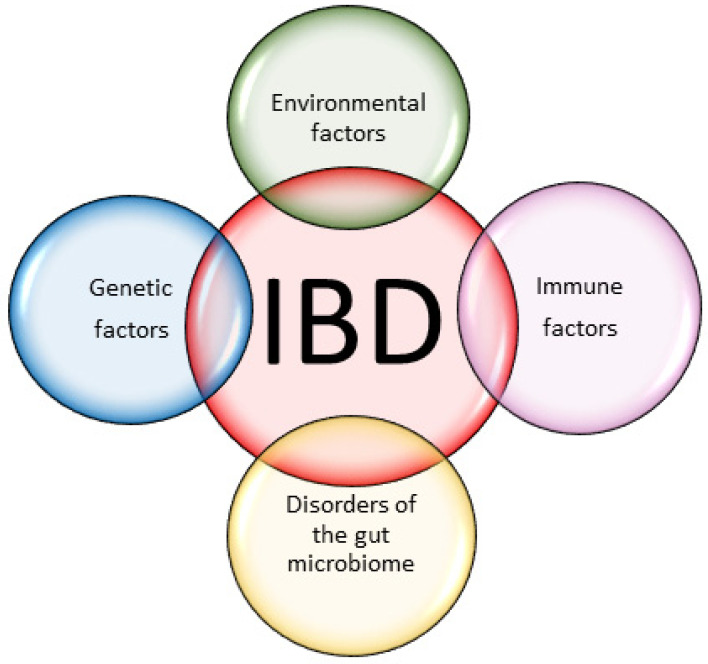
Factors affecting the development of IBD.

Many factors are responsible for the development of ulcerative colitis and Crohn’s disease. Environmental factors are the main cause of the increase in IBD in people with a genetic predisposition or immune disorders, but it is the coexistence of several factors simultaneously that is responsible for the development of the disease. The immune system of the intestinal mucosa provides a protective barrier to the integrity of the gastrointestinal tract, loss of intestinal epithelial barrier function leads to excessive bacterial translocation, which also contributes to the development of IBD [25]. Figure 3 shows factors affecting of IBD.

### 2.4. Genetic Factors

It is widely known that the risk of IBD is higher among affected family members, with twins having the highest risk, followed by first-degree relatives [26]. To date, more than 201 sites in the genome that determine IBD susceptibility have been identified [27]. Analysis of genes involved in IBD shows that multiple pathways may be responsible for the development of the disease, where the intestinal barrier appears to be the most important. Genome-wide association study (GWAS) is one method to analyze the prediction of IBD disease. The genes involved in the development of IBD are summarized in Table 4 [28,29].

Some genetic disorders have been found to be specific to particular disease. For example, genetic studies conducted have shown, among other things, *NOD2*, *ATG16L1*, and *IRGM* mutations leading to chronic IBD. *NOD2* mutation leading to over activation of the MAP pathway causes chronic intestinal inflammation [30,31]. Mutations in *ATG16L1* and *IRGM* have been linked to autophagy disruption in Crohn’s disease, which is responsible for the removal of degraded proteins and mitochondria, which plays a key role in innate and acquired immunity [32,33,34,35]. Disruption of *ECM1* encoding extracellular matrix and activating NF-κB signaling and Il-10 gene defect causing its defective function are associated with CU [36,37,38]. The first genotype–phenotype association studies were conducted on IBD subphenotypes and HLA variability [39,40,41,42,43,44]. These studies were mostly limited in number of patients (approximately 100 patients) and showed mixed results. Overall, the common results showed a correlation between HLA alleles and extensive colitis and colectomy in UC. Additionally, HLA DRB1*01:03 is also associated with localization of colon disease in CD [45,46]. *NOD2* is the first gene associated with CD. Several genotypic–phenotypic studies involving several hundred subjects have attempted to link *NOD2* variants to CD subphenotypes [47,48,49,50,51,52,53]. The results of HLA studies were also mixed, and most often related to associations with *NOD2* variants for colorectal disease, narrowing behavior and younger age. An impressive study from the above scope was the study of IBD chips using a custom chip that included the whole genome and nominally relevant replicated loci from the meta-analysis [54]. They were used in this study to investigate their impact on clinical outcomes in 1528 patients with CD [54,55]. The results showed that three genes were associated with multiple subphenotypes. *NOD2* has been associated with colorectal disease, constricting, penetrating behavior, need for surgery, and disease. Localization of colorectal disease and narrowing behavior were also associated with *JAK2*, while penetrating behavior and complicated disease were associated with *PRDM1*. Variants that are associated with subphenotypes do not necessarily have to be identical compared to variants associated with the development of IBD. One study included 1762 CD patients with poor prognosis, often exacerbating refractory disease, and 972 CD patients with good prognosis and slow disease [56]. Four loci—*FOXO3*, *MHC*, *XACT*, and one near *IGFBP1*—were found to be relevant to the whole genome. Cleynen et al. conducted the largest genotype–phenotype study ever conducted by the International Inflammatory Bowel Disease Genetics Consortium and relied on immunochip data in 16,902 CD patients and 12,597 UC patients [57]. The subphenotypes studied were age at diagnosis, time to surgery, disease location (CD), disease behavior (CD), and disease grade (UC). The analysis included 156,154 genetic variants, but only three loci (*MST1*, *NOD2*, and *MHC*) showed important genomic-wide associations with one or more subphenotypes. All three loci were associated with age at diagnosis as well as disease location (CD). Time to surgery was associated with MHC in all IBD patients and with *NOD2* in CD patients. However, the researchers focused only on the variants available in the immunochip, a genotyping chip, which is intended for immunogenetic studies. However, in a more recent study, imputed data referring to the genotype from the broader SNP array were used. It was enrolled in 1495 patients with CD [58]. But it has not been possible to identify any significant linkages that span the whole genome with time to surgery, time to disease progression, or disease of slow or progressive course. Innovative therapies that target specific genes or gene products involved in the pathogenesis of the disease have given the opportunity for individualized treatment [59,60,61]. Recently, approximately 200 loci susceptible to IBD have been identified, corresponding to different factors (patient age, ethnicity, and race) [62,63,64,65]. Although many genes associated with IBD have been studied, only a dozen or so have been well understood and defined as genes essentially associated with IBD and 70% of them are associated with Crohn’s disease and ulcerative colitis [63,66,67]. These genes fall into three categories that act on different control points of the inflammatory pathway. These categories are: 1. pathogen recognition, 2. pathogen removal by innate as well as cellular immunity, and 3. obstruction of pathogen invasion through the intestinal mucosa barrier [68,69]. A healthy person may be screened positive for an IBD mutation without any clinical or histological evidence suggestive of IBD [70]. This highlights the importance of additional factors in the development of the disease [61,63,67,70,71]. Age of onset of IBD varies depending on the genetic profile and the presence of external factors (environmental, nutritional, and social factors) [70,71].

### 2.5. Biomarkers

Several biomarkers have been studied, focusing on the degree of correlation with UC. The most commonly used biomarkers are: ESR, C-reactive protein (CRP), fecal calprotectin (FC), and also fecal lactoferrin (FL). OB and CRP markers are helpful in identifying inflammatory and non-infectious causes of diarrhea but they are non-specific markers whose levels are usually elevated in certain disease states. The ESR marker, on the other hand, is a nonspecific marker and does not change as rapidly as CRP. This results in its limited utility [72,73]. Significantly, studies conducted as recently as the late 1990s, showed that approximately 50% of patients with active disease may not have elevated CRP levels [74,75]. FC and FL are more specific markers for inflammatory bowel disease and are more closely associated with colonic disease [76,77]. Researchers Chen, Shang et al. identified 10 hub genes that are associated with pyroptosis in UC. In addition, they verified the gene expression pattern of these hub genes. The effect of existing drugs used to treat UC on the expression of hub genes was investigated. IL1B, a predictor of drug response and also a marker of active UC status, was identified. After combining single-cell analysis along with immune infiltration, macrophages were identified as the most relevant immune cell type throughout UC progression. In addition, they also investigated the molecular mechanisms of the process of pyroptosis in UC. The results showed that crosstalk between macrophages and IECs that relate to pyroptosis may affect the unrelatability and recurrence of UC. Thus, the resulting chain of 1B-macrophage-pyroptosis relationship may provide new insights into the pathogenesis and also the treatment of UC [78]. Another group of researchers from the American Gastroenterological Association made seven conditional recommendations for UC. For patients with the disease in symptomatic remission, the researchers suggest using a monitoring strategy that is based on biomarkers or symptoms instead of a monitoring strategy that is based on symptoms. Patients in symptomatic remission should have fecal calprotectin <150 µg/g, normal fecal lactoferrin, and/or normal CRP levels to rule out active inflammation and avoid routine endoscopic evaluation of UC. UC patients with moderate to severe symptoms should have either fecal calprotectin >150 µg/g, elevated fecal lactoferrin, or elevated CRP. Researchers identified the use of a biomarker-based monitoring strategy instead of an endoscopy-based monitoring strategy as a knowledge gap in the field [79].

### 2.6. Imaging Methods

Several imaging methods have been investigated to assess disease activity in UC. Intestinal ultrasound was studied using endoscopic ultrasound probes and with transabdominal access [80,81,82,83]. While studies have shown that the first method is more accurate, the usefulness of endoscopic ultrasonography is somewhat limited, due to the fact that adequate preparation of the intestine is necessary and this study has an invasive nature. Transabdominal access has also been shown to be well correlated with Mayo 2 endoscopic disease and continues to be an area of research and interest. The impact of MRI was also studied [84,85]. However, few protocols have the advantage of not requiring bowel preparation combined with faster imaging. Thus, they have been shown to correlate well with endoscopic results [84]. Despite the results obtained, the use of these imaging methods in the assessment of CD disease as well as UC in the United States is slow.

### 2.7. Environmental Factors

The earliest documented environmental factor influencing IBD was smoking [86,87]. However, smoking protects against ulcerative colitis [88]. These findings highlight the complexity of environmental influences in IBD. The main environmental factors are shown in Table 5 and Table 6 [89,90].

As information on IBD has developed, many environmental factors have been linked. A change in intestinal microflora (dysbiosis) is associated with the onset or progression of IBD. Early childhood events (birth, breastfeeding, and antibiotic exposure) or later childhood events such as potential risk factors for IBD. In addition, air pollution, i.e., the consequence of the progressive contamination of the environment by a large number of compounds, is another factor associated with IBD. This is because solids or other components may affect the host mucosal defense mechanisms and often trigger immune responses [91,92,93]. Environmental factors have a significant impact on the understanding of IBD pathogenesis. In addition, they define that the disease requires complex therapies, which now go far beyond the one-way treatment approach [94].

### 2.8. Immune Factors

**Figure 4:**
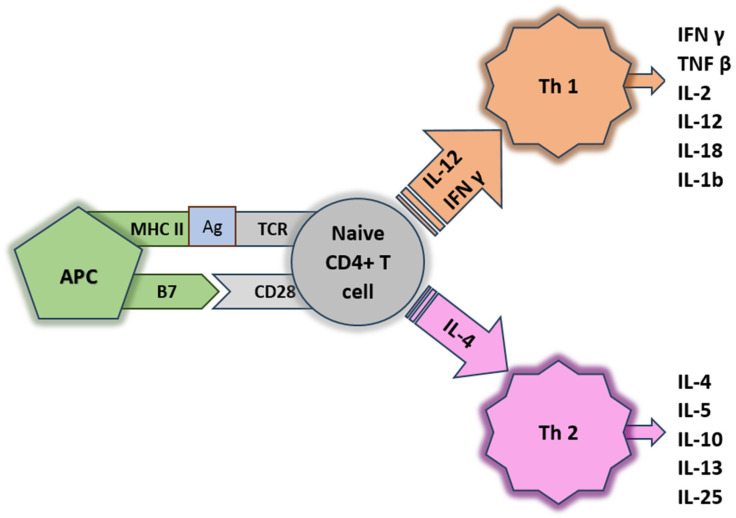
Scheme of cytokines production. APC: anaphase-promoting complex, CSF: colony stimulating factor, EBI: Epstein–Barr virus-induced gene, IFN: interferon, IL: interleukin, LIF: leukemia inducing factor, LT: lymphotoxin, OSM: oncostatin M, TGF: transforming growth factor, TL1A: TNF-like cytokine 1A, TCR: T-cell receptor, TNF: tumor necrosis factor, TNFSF: TNF super family member, TSLP: thymic stromal lymphopoetin. Genome-wide association studies have identified several IBD susceptibility loci containing genes encoding cytokines as well as proteins involved in cytokine signaling. Mutations that cause loss of function in genes encoding interleukin-10 (IL-10) and the IL-10 receptor are associated with very early onset IBD [97].

Studies focusing on characterizing the host immune response in IBD indicate that in CD the response is associated with activation of Th1 cells, while in CU the response is associated with a Th2 cell population. Lymphocytes with a Th1 phenotype are responsible for the production of cytokines such as IL-2, IL-12, IL-18, interferon gamma, IL-1b, and TNF alpha. Lymphocytes with a Th2 phenotype are primarily responsible for the production of IL-4, IL-5, IL-10, and IL-13; see Figure 4 [95,96,97].

### 2.9. Characterization of the Most Important Cytokines in IBD

The study of the immune response has led to the observation that the two main types of IBD are distinct forms of enteritis, that is, CD is induced by the Th1 response, and UC is associated with unconventional enteritis [94,98]. Like Th17 cells, it is also involved in the inflammatory bowel response in IBD [99].

The innate immune response plays an important role in defending against pathogens. It is mediated by many different cell types (epithelial, neutrophils, dendritic cells, monocytes, macrophages, and Natural Killer cells (NK cells)) [100]. This type of immunity is initiated by the recognition of microbial antigens through receptors that recognize patterns, including Toll-like receptors (TLRs) on the cell surface and NOD-like receptors found in the cytoplasm [101]. Studies show that the behavior of cells, which are responsible for innate immunity and the expression and function of TLR and NOD proteins undergo tremendous changes in IBD patients. Mucosal neutrophil accumulation and injury-related IL-1β and IL-8 production are reduced in CD patients but not in UC patients [102].

According to GWAS, *NOD2* mutations often associated with CD result in a defective ability of the intestine to respond to LPS. Consequently, this gives a defect that can affect susceptibility to diseases [103,104]. The role of the *NOD2* mutation remains controversial as current evidence suggests that there are mutations that cause loss of function leading to less NF-κB activation [104]. In combination with the above, an insufficient response may affect reduced production of antibacterial agents as well as invasion of pathogenic micro-organisms [105]. There are also studies that indicate that loss of *NOD2* function may lead to a lack of inhibition of TLR2 stimulation, resulting in activation of inflammatory pathways and excessive Th-1 responses [106]. *NOD2* also contributes to immune tolerance. However, these effects are significantly attenuated in cells of patients who have the *NOD2* 3020insC mutation [107]. IL-23 is a key cytokine in both innate and acquired immunity, as it plays an important role in eliciting early anti-microbial responses. *IL23R* polymorphisms are related to both CD and UC. Thus, IL-23 may represent a common inflammatory molecule in chronic enteritis. In addition to acting on Th17 cells, IL-23 may also act on cells of the innate immune system. Studies have shown that IL-23 induces the production of Th17 cytokines by congenital lymphoid cells having a lymphatic tissue-induced cell phenotype [108] (Table 7).

**Figure 5:**
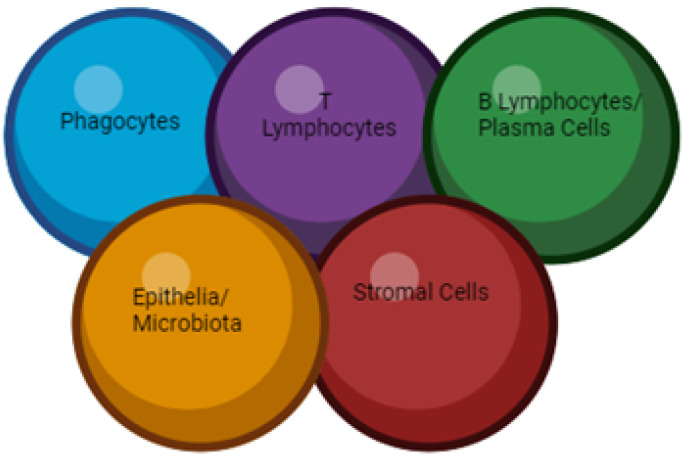
Cytokinases and their interrelations.

West et al. have developed relative levels of expression in transcriptomic datasets [109] and produced a table of cytokines involved in CD, UC, or both disease units [109,110,111,112,113,114,115,116,117,118,119,120,121,122,123,124,125,126,127,128,129,130,131,132,133,134,135,136,137,138,139,140,141]. In transcriptomic assays, a large proportion of cytokines are not clearly regulated. However, the lack of regulation in the tissue that is affected by inflammation does not exclude it from the pathogenesis of IBD. Cytokines may act in specific immune compartments not subject to endoscopic examination [142]. Figure 5 shows the modulated cytokines placed in the context of the inflammatory response in IBD. As you can see, all the following groups of cytokines overlap, and it is clear that they are interrelated. These cytokines have different functions, but they can be grouped into modules related to phagocytes, T lymphocytes, B lymphocytes, and plasma cells, as well as regulators of epithelium, microflora, and stem cells [143].

IFN-gamma is one of the most important cytokines that participates in the induction and modulation of a variety of immune responses in the human body, including through the activation of macrophages and it is characterized by antiviral, immunoregulatory, and anticancer activity. Interferon gamma was first described in 1965 by E. Frederick Wheelock as an interferon-like virus inhibitor that was induced by Phytohemagglutinin derived from an extract of the Phaseolus vulgaris [144]. IFN gamma is the only member of type II interferons whose human gene locus is located at 12q14þ3 chromosome. IFN-gamma combine with the receptor, which is formed by two subunits *IFNGR-1* and *IFNGR-2*. Interferon gamma is now known to participate in the signal transduction pathways, mediating immune responses [145]. The role of IFN-γ as an initiator of the inflammatory process in the intestines has been demonstrated in several mouse models. In vivo studies have shown that in people with high production of IFN-gamma, intestinal damage occurred after exposure to toxic factors compared to people with a deficiency of this cytokine [146,147]. IFN-γ as an immunomodulatory exerts strong effects on the vasculature, because IFN causes vascular barrier breakdown by disrupting VE-cadherin protein of the adherens junction. This was used in the Langer et al. study, where restoration of vascular barrier function was achieved and inflammation induced by DSS was reduced after imatinib treatment [148]. Woznicki et al. also showed that TNF-α synergises with IFN-γ to induce enzyme dependent death of intestinal epithelial cells [149]. IFN gamma have been shown to disrupt epithelial barrier integrity both in vivo and in vitro in the study by Madara et al. [150].

IL-4 regulates antibody production and hematopoiesis, plays a key role in inflammatory reactions, and has mitogenic activity in endothelial cells [34,35]. Other properties of IL-4 are its Th2 and Th1 immune functions, in which it can initiate or inhibit a given reaction. Increased secretion of IL-4 by Th2 cells is characteristic in colitis ulcerosa [151,152]. Studies also suggest that induction of an IL-4-dependent immune response may be a major pathogenic factor in ulcerative colitis exacerbation [153]. IL-6 is a pleiotropic cytokine with roles in immunity and metabolism. Interleukin 6 inhibits the reverse secretion of TNF alfa. Interleukin 6 stimulates B lymphocytes to release immunoglobulins of different classes, stimulates synthesis of acute phase proteins in the liver and phospholipase A2, stimulates bone marrow progenitor cells and platelet production, regulates metabolism, and stimulates bone resorption [154].

The study by Gross et al. showed that IL-6 is higher in patients diagnosed with Crohn’s disease. Studies analyzing the concentration of selected interleukin were performed from collected serum and intestinal mucosa [155]. Additionally, the authors confirmed that the concentration of Il-6 depends on the progression of the disease. Additionally, it correlates with the frequency of relapses and with inflammatory symptoms of the disease [156].

In turn, interleukin 10 (IL-10), as an anti-inflammatory cytokine, has inhibitory properties, inhibiting the production of many cytokines, e.g., proinflammatory cytokines. According to the literature, IL-10 has properties that regulate various types of innate and adaptive immune cells. This treatment helps avoid the development of various immunological pathologies, which include cell induction and autocrine inhibitory effects on macrophages and dendritic cells [157]. Low levels of interleukin IL-10 may cause prolonged activation of mononuclear cells, thereby increasing the production of inflammatory cytokines. This phenomenon may lead to, among other things, damage of the intestinal mucosa [158]. The basic principles of anti-cytokine therapy in IBD are based on the use of anti-inflammatory agents, aminosalicylates, and corticosteroids [158]. Additionally, a large number of studies have investigated potential beneficial effects of anti-cytokine antibodies in IBD patients. Infliximab was the first antibody in IBD therapy [159]. Adalimumab was shown to be effective in IBD patients in both CD and UC [160]. TNF-receptor (TNF-R) fused with a Fc domain of human immunoglobulin (Ig) G1, that binds and inactivates TNF, failed to show a clinical benefit in CD patients [161]. Sirukumab, olokizumab (CDP6038), C326, PF04236921, and BMS-945429, as well as tocilizumab targeting the IL-6 receptor have been investigated in IBD patients [161].

## 3. Characteristics of the Main Indicators of Inflammatory Bowel Diseases: Metalloproteinases: MMP-3, -7, -9, and -11

The focus on the aforementioned metalloproteinases is based on the fact that they have been the subject of our research from the very beginning; hence, the characterization of only these metalloproteinases. In IBD disease, the pathological process is associated with extensive degradation of the mucous membrane, as well as with tissue remodeling, which promotes the development of ulcers, fistulas, and narrowing. The pathogenesis of the aforementioned changes is not yet well understood, and many studies confirm the involvement in these processes of a large number of proteases, which are produced in inflammatory microenvironments. These include, for example MMP [162].

MMPs are primarily secreted as latent, inactive zymogenes by a large number of different cells (e.g., myofibroblasts, T cells, macrophages, monocytes, neutrophils, and epithelial cells). Conversely, conversion to the active enzyme occurs most often in the pericellular or extracellular space. MMPs are characterized by the fact that they act together, forming an activation cascade. This process works in such a way that when activated, one MMP can induce the conversion of other MMP zymogens to their active forms, forming a catalytic cascade that has the ability to degrade many components proteoglycans, collagens, and non-collagen glycoproteins [162]. Considering the primary substrate, MMPs are divided into subclasses, which are collagenases, gelatinases, stromelysins, elastases, membrane types, and others. The classifications of the above mataloproteinases are listed below (Table 8).

Studies by Saarialho-Kere et al. have documented high expression of MMP-1 and MMP-3 RNA in gastrointestinal tissue around ulcers, also in the intestines of patients with IBD [163]. Subsequently, the same researchers showed that MMPs are produced by different types of cells in the gut—laminino-5-positive and Ki67-negative enterocytes that surround ulcers expressed MMP-10 mRNA. Macrophages near the exfoliating mucosal epithelium and below the necrotic surface of ulcers were positive for MMP-12 and fibroblast-like cells in ulcers were the source of MMP-13 [164]. Dobre et al. documented increased expression of MMP-10 RNA in inflammatory tissue of UC patients [165]. Other studies have confirmed increased RNA expression of many MMPs in tissue that is inflamed in CD and UC patients compared to healthy and diseased controls (e.g., diverticular disease) (Table 8) [166]. In the pediatric population, MMP-7 expression was significantly pronounced in active CD as compared to active UC. This gave hope that the MMP-7 could be helpful in distinguishing a CD from a UC [167]. Another study in this regard confirmed increased expression of MMP-7 with MMP-1, -3 and -10 in the area of intestinal epithelial cells and CD and UC mononuclear plaque cells (LPMC) [168]. It was also noted that MMP-1 remained elevated in UC patients with endoscopic remission with persistent histological inflammation [169]. MMP-1, MMP-3, and MMP-9 are largely produced by mucosal myofibroblasts [170] and have also been observed in the fistula tract [171,172] while neutrophils produced MMP-2 and MMP-9 [173]. On the other hand, epithelial cells on the margins of ulcers most often produce MMP-7 [174,175] This study indicates that MMP-7 plays roles in the re-creation of epithelium after injury. In addition, expression of the MMP-9 gene and protein was particularly increased in patients with extensive UC compared to patients with left-colonic lesions or healthy controls [176]. IBD is characterized by increased plasma concentrations of various MMPs [177,178]. However, there is no clear evidence to support the use of circulating MMP as an indicator of disease activity. In IBD, an excessive immune response is associated with abnormal production of several MMPs as well as altered MMP/TIMP ratios. Studies confirm the role of MMP in the process of mucosal degradation, which is associated with IBD. Several MMP inhibitors have been developed and used to alleviate enteritis in animal models of IBD [179]. Similar results were also seen after treatment with batimastat, which is an MMP inhibitor [180]. Studies by O’Sullivan et al. show that rectal administration of diazotane-barbiturate, a barbiturate-based MMP inhibitor that includes a group of nitric oxide donors/mimetics, to rats with colitis with sodium dextran sulfate (DSS) inhibits the induction and activity of MMP-9. In addition, it relieved the ongoing colitis [181]. The above data allowed the development of compounds for clinical use. Three clinical trials have been conducted with MMP inhibitors for the treatment of IBD. The first phase of the study focused on the safety and efficacy of GS-5745 (andecaliximab), a fully humanized, high-affinity IgG4 monoclonal antibody that selectively binds to and inhibits MMP-9 in the moderately to severely active form of UC, with very impressive results [182]. Seventy-four UC patients were randomized to receive either a single or multiple initial intravenous dose (0.3, 1.0, 2.5, or 5.0 mg/kg) two weeks apart (three infusions) or five weekly subcutaneous doses (150 mg) of GS-5745 or placebo. The medicine was safe and 43% of patients who received GS-5745 had a clinical response compared with 13% of patients who received placebo. However, these results were not confirmed in the phase 2 and 3 studies [183]. GS-5745 was tested in phase II in patients with moderate to severe CD. However, eight weeks of induction treatment did not result in adequate symptomatic or endoscopic responses [184].

### 3.1. MMP-3

Matrix metalloproteinase-3, or MMP-3, is one of the representatives of the stromelysin group [46]. MMP-3 plays a key role in many processes, both physiological and pathological. It is present in processes such as tissue morphogenesis, inflammatory reactions, and damage repair. It has proteolytic properties, so it has the ability to destroy chondrocytes. Additionally, it is involved in the initial phase of rheumatoid arthritis [185]. MMP-3 expression influences the ability of many types of malignancies to degrade ECM, e.g., glioma, breast cancer, liver cancer, etc. [186,187,188,189,190]. In addition, Kahlert et al. showed that colorectal cancer cells have abnormally high expression of MMP-3 protein [191]. MMP-3 may play a key role in colon cancer growth and migration promoted by collagen degradation, according to recent studies [192,193]. MMP-3 matrix lysin that digests ECM components [185]. In their study, Sipos et al. found a positive correlation between increased expression of the MMP-3 protein and the adenoma–dysplasia–cancer process [194]. The dysplastic states of established adenocarcinoma, which are characterized by high-grade malignancy and early-stage CRC, can be distinguished by MMP3 expression in the stroma [194]. A study by other researchers showed a positive relationship between the level of MMP-3 protein expression and lymph node metastases [195]. Additionally, it has been shown that MMP-3 can activate other metalloproteinases, such as MMP-1, MMP-7, and MMP-9, mainly to activate cancer cell division [172,196,197,198,199,200,201,202]. Kirkegaard et al. in their work noted high concentrations of MMP-3 in the fistula tissue of patients with Crohn’s disease compared to the control group in which inflammation was not diagnosed. The analysis detected the presence of metalloproteinase in mononuclear cells and fibroblasts as well as in fistulas [172]. MMP-3 has also been identified in idiopathic fistulas. Lièvre examined [203] gene promoter polymorphism mainly in MMP-3, but also in MMP-7 and MMP-1 in patients with adenomas. The experimental results confirmed a close and strong correlation between MMP-3, MMP-1 polymorphisms, and adenomas. No such relationships were found with MMP-7. This type of research plays a significant role in analyzing the origin and activity of adenomas [202]. Another study assessed the role of cytokines and metalloproteinases, which play an essential role in colitis. These genes include: MMP-3, MMP-9, MMP-7, and MMP-13. They can be used as a kind of natural molecular markers in assessing the extent of inflammation [192]. In turn, Pan et al. [204] analyzed genes, cytokines, and metalloproteinases associated with colon diseases, i.e., ulcerative colitis and colon cancer. In the study, they confirmed that patients diagnosed with an active form of ulcerative colitis had higher concentrations of MMP-3. In turn, after the administration of golimumab, there was a decrease in the expression of not only the concentration of MMP-3 and TIMP-1 in the mucosa of patients. Similar correlations have been observed in patients with colorectal cancer [203]. Scientists have confirmed that MMP-3 is a promising marker of inflammatory bowel diseases. Similar conclusions were presented by Li et al. [205], who recruited and examined 260 patients in 2016–2020. Of the 35 cytokines identified, MMP-3 and CC2 were the two most effective serum biomarkers [204]. Innate lymphoid cells (ILC) and their cytokines may play a central role in the pathogenesis of IBD, especially ILC3 (UC) and ILC1 (CD) [205]. The role of neutrophils, including neutrophil extracellular traps, and various types of macrophages (M) and NK/NKT can also be noted. In addition to IFN-γ, interferons—IFNL also play an important role in the pathogenesis of CD [206]. The theory that cytokines in UC IL-22, IL-17 (Th17, ILC3), IL-22 (Th17, Th22), and IL-9 (Th-9) under pathological conditions (e.g., IBD) may cause epithelial inflammation due to endoplasmic reticulum (ER) stress response is worth further investigation [207,208]. Table 9 presents the pathogenetic role of cytokines in UC [209,210,211].

### 3.2. MMP-7

MMP-7 is a metalloproteinase and proteolytic enzyme that produces zinc and calcium endopeptidases [205]. Its main function is a regulatory role in various pathophysiological processes. For example, it participates in the immune response in co-operation with other elements of the immune system [205]. Additionally, it activates cryptins, i.e., antimicrobial peptides [2]. This metalloproteinase may be a promising biomarker of cancer and, also, a therapeutic target. MMP-7 expression is associated with clinical characteristics of cancer patients [211,212]. Manipulation of MMP-7 expression or function may become a potential treatment strategy for various types of diseases, most notably cancer [213]. There are four characteristic regions in the structure of MMP-7, but the hemoglobin terminal group is missing. Normally, MMP-7 metalloproteinase is present in high concentrations in organs such as bronchioles, epithelial tissues of skin glands, and the gastrointestinal tract [214,215]. It is present in small amounts in the lungs, gallbladder, and urinary bladder. When inflammation or disease begins, their levels increase [216,217,218,219,220]. MMP-7 is also responsible for the effective wound healing process and participates in the processes of various signaling pathways responsible for cell growth and angiogenesis [221,222,223,224,225,226]. MMP-7 is expressed in human multi-organ cancers [227,228], including gastrointestinal cancers [227]. Additionally, MMP-7 may act as an oncogenic protein that regulates the physiology of various cancers. According to the available literature data, it can be concluded that the level of MMP-7 [167,174,175,229,230] is increased in inflammatory tissues. In patients with colorectal cancer, MMP-7 is responsible for cell proliferation [231,232,233,234,235,236] by releasing ectodomains, i.e., growth factors [234,237]. In turn, Klupp et al. [235] analyzed the level of MMP-7 in serum samples of patients diagnosed with colorectal cancer. Studies have shown that the level of MMP-7 was higher in patients with colorectal cancer compared to the level of MMP-7 in patients from the control group, i.e., healthy patients [234]. Overall survival rates were also analyzed, which were lower in patients with colorectal cancer compared to patients from the control group [232]. High concentrations of MMP-7 are responsible for excessive proliferation of cancer cells and, consequently, contribute to the metastasis of cancer cells (mainly in the case of colorectal cancer) [231]. Therefore, scientists claim that the analysis of MMP-7 and its determination in tissues and serum can be used as an independent prognostic indicator in the case of the large intestine [232]. Both MMP-3 and MMP-7 in cancer cells may or may not determine tumor resistance to apoptosis [218,219,220,221,222,223,224,225,226,227,228,229,230,231,232,233,234,235,236,237,238,239,240,241,242]. Some MMPs, including MMP-3 and MMP-7, are involved in the transport of cancer cells.

### 3.3. MMP-9

MMP-9 also plays a significant role in the progression of cancer cells. According to a review, it is one of the most frequently studied MMPs [238,243]. According to Daniluk et al., MMP-9 is a marker of destruction (to a greater or lesser extent) of the intestinal mucosa, mainly in Crohn’s disease [244]. In the experiment, the research group and the control group were pediatric patients. The experiment used an immunoassay. The results confirmed previous hypotheses that MMP-9 levels were higher in patients with Crohn’s disease compared to the control group. According to the authors, increased MMP-9 concentrations are a reliable marker of inflammation, especially in Crohn’s disease [239]. The analysis of MMP-9 concentrations is also used in systemic diseases, e.g., thromboembolism [184,240,241,242,243,244,245,246,247,248,249]. A similar study was conducted by the research group in Kofla-Dubacz et al. [250]. They assessed the concentration of MMP-3 and MMP-9 in Crohn’s disease. They examined the correlation between MMP-3 and -9 concentrations and clinical disease activity. As in the previous case, in this experiment the research group was a group of pediatric patients diagnosed with Crohn’s disease. The results confirmed that the concentration of MMP-9 in serum correlates and is dependent on disease activity [245]. Similar research was conducted by Piechota-Polańczyk et al. [251]. The aim of the study was to analyze the correlation between cyclophilin A and MMP-9 in inflammatory and non-inflammatory conditions of the large intestinal mucosa in patients with Crohn’s disease. The subject of the study were serum samples and tissue of the large intestine mucosa taken during biopsy. In this experiment, ELISA was performed. And in this case, the results were not as good as in the previous ones. Higher MMP-9 concentrations were detected in patients with Crohn’s disease [246]. Similar results were presented by Meijer et al., who showed increased activity of matrix metalloproteinases in tissues associated with inflammatory bowel disease [41]. In turn, Gao et al. and de Bruyn et al., attempted to assess the effect of infliximab therapy on MMP-9 expression in Crohn’s disease. The authors observed that the level of MMP-9 decreased after the use of infliximab [252,253]. An example of a Polish research team that estimated the concentration of MMP-9 in the serum of patients with inflammatory bowel diseases was the team of Matusiewicz et al. In the results, the authors presented that the concentrations of MMP-9 in the serum were significantly higher in the active phases of the disease. The authors agree that the assessment of MMP-9 concentration in serum may help in the differentiation of Crohn’s disease [254]. In studies conducted by Siloşi et al. [255] and Mäkitalo et al. [256] the aim was to examine the content of MMP-9 in the stool of patients with inflammatory bowel disease. MMP-9 concentrations were also analyzed by ELISA. The experiment confirmed that MMP-9 levels were significantly higher in cases of active intestinal inflammation and ulcerative colitis [255]. Mäkitalo et al., [256] assessed the concentration of MMPs and their inhibitors in the serum of pediatric patients with IBD after pharmacotherapy. In this case, the experimental part was also performed using the ELISA test. As a result of the experiment, it was observed that the concentration of MMP-9 in the serum before treatment was increased compared to the control group.

Another research team assessing the expression of MMP-9 in ulcerative colitis and Crohn’s disease is the team of scientists Jakubowska et al. [257]. The assessment of expression in tissue samples was performed using the immunohistochemical method. Experiment showed that MMP-9 overexpression predominated in both the glandular epithelium and the inflammatory infiltrate. The study confirmed that MMP-9 may be a potential therapeutic target in inflammatory bowel diseases.

Another group of researchers examining the relationship between serum MMP-9 levels and disease activity in IBD patients was the group of Shamsey et al. [258]. The study observed that serum MMP-9 concentrations were higher in patients with active ulcerative colitis compared to patients with inactive disease. No elevated values were observed in the control group either. Serum MMP-9 levels were also higher in patients with active Crohn’s disease compared with patients with inactive disease. The authors concluded that the measurement of MMP-9 in serum can be used to differentiate active and inactive stages of the disease [258]. The next research group was that of Yablecovitch et al. [259]. The aim of their study was to evaluate whether serum MMP-9 levels predict clinical exacerbation in patients with Crohn’s disease. Higher MMP-9 levels were found in patients who later experienced disease exacerbation. In summary, the authors demonstrate that MMP-9 may be a promising marker for predicting exacerbations of the clinical phase of Crohn’s disease [259].

### 3.4. MMP-11

In turn, MMP-11 from the endopeptidase group is involved in matrix degradation and tissue remodeling. Currently, there is a belief that MMP-11 promotes cancer development. Compared to other MMPs, MMP-11 cannot have a destructive effect on any of the components of the extracellular matrix. Additionally, MMP-11 is secreted in its active form. MMP-11 participates in tissue remodeling processes, including those related to cancer progression [260]. MMP-11 is an important protease that is expressed in cancer cells, stromal cells, and the surrounding microenvironment. MMP-11 has a bilinear effect on cancer. On the one hand, it supports tumor growth by inhibiting apoptosis and promoting cancer cell migration. On the other hand, in animal models, MMP-11 has a protective effect on tumor growth and metastasis at more or less advanced stages [261,262]. Huang et al. [263] conducted research to determine the levels of MMP-9 and MMP-11. The experimental results confirmed that the concentration of MMP-9 and MMP-11 was higher in patients with colorectal cancer compared to the concentration level in healthy patients. The results showed that the combined detection of metalloproteinases in serum can be a specific and sensitive diagnostic biomarker [263]. Increased serum levels of MMP-11 are observed not only in inflammatory tissues of IBD but also in cancerous tissues of the stomach, breast, and pancreas. Currently, research confirms that MMP-11 may be a prognostic factor for detecting early-stage cancer. Additionally, it can help assess the degree and extent of the cancer. Currently, there is a need to conduct further research analyzing the role of MMP-11 in cancer progression [264,265]. Arcidiacono et al. [266] investigated the expression of MMP-11 in adipose tissue dysfunctions using in vitro and in vivo models of insulin resistance. The research was conducted in laboratory conditions on mice. The results showed that MMP-11 mRNA expression levels were significantly higher in insulin-resistant adipocytes compared to control cells. It is worth noting that the results obtained in in vitro experiments were confirmed in an in vivo model of insulin resistance. The authors conclude that dysregulation of MMP-11 expression is the initial stage of the process of adipose tissue dysfunction, which may, consequently, lead to problems with insulin resistance [266]. Zhang et al. [267] conducted research to confirm the influence of MMP-11 in tumorigenesis. The authors analyzed the possible mechanism of tumor initiation in patients with pancreatic cancer. The results showed that MMP-11 could be expressed. The site of its activation was the cytoplasm [267]. In turn, Motrescu et al. [268] presented a short review in which they analyzed the role of MMP-11 in the context of cancer formation. It is worth noting that MMP-11 plays a significant role during tumor desmoplasia and constitutes a molecular link between obesity and cancer [268,269]. MMP-11 also plays a significant role in hepatocellular tumor migration and metastasis [270]. There are also examples in the literature of the characterization of MMP-11 in prostate cancer. It is involved in the degradation and remodeling of the extracellular matrix and plays an essential role in the development of prostate cancer and metastasis. Studies show that it may contribute to the development of cancer in prostate cancer patients with biochemical recurrence [271]. Another example of assessing MMP-11 expression is cholangiocarcinoma, which is a primary tumor of the bile duct mucosa. The aim of the work by Tongtawee et al. [272] was to detect the expression of MMP-11 in samples and show the relationship with survival time. The research group consisted of 30 patients who underwent MMP-11 immunohistochemical staining. In the results, the authors showed that MMP-11 expression was found in 50% of patients. Overall median survival time was 237 days. The authors concluded that positive expression of MMP-11 indicates a poor prognosis in samples with cholangiocarcinoma [272]. Another example of a condition where researchers are evaluating the relationship between serum MMP-11 levels and patient prognosis is colon cancer. The article by Pang et al. [273] analysed MMP-11 levels in the serum of patients with colon cancer. The experiment examined the associations between serum MMP-11 levels and clinico-pathological characteristics of patients. In the results, the authors showed that serum MMP-11 levels were higher in patients with colorectal cancer compared to healthy control patients. According to the authors, high levels of MMP-11 in serum correlated with poor clinical outcomes [268]. Nakopoulou et al. [274] conducted studies characterizing MMP-11 in various types of glomerulonephritis. In the results, the authors showed that immunopositivity for MMP-11 was detected in the glomeruli of most patients. The highest incidence of MMP-11 was reported in glomerulonephritis [274]. The MMP-11 biomarker is used to diagnose and predict bladder cancer.

Studies on the analysis of MMP-11 in bladder cancer were also conducted by Chen et al. [275]. In this study, they analyzed the expression of MMP-11 in patients with bladder cancer. In this study, they confirmed that increasing MMP-11 levels is associated with tumor progression and poor survival in bladder cancer patients. The presented results suggest that MMP-11, as a secreted protein, is a promising biomarker in the diagnosis and prognosis of patients with bladder cancer [275].

## 4. Summary

Metalloproteinases correlate with inflammation, signaling the phase of the disease [125]. Analysis of MMPs concentration is also useful in inactive disease states. In this case, a biochemical test is performed to confirm inflammation [153]. A similar observation was made by Bouma et al., [41] who also assessed inflamed tissues using biochemical analysis of mucosal metalloproteinase activity in patients with Crohn’s disease. They characterized the markers MMP-1, -2, -3, and -9. Inflammatory tissues showed increased activity of all 4 metalloproteinases, which could have influenced changes in tissue morphology and physiology [154]. Moreover, MMP-7 is a biomarker of Crohn’s disease as a marker differentiating inflammatory tissues. In the study by Rath et al. [175,230] increased MMP-2, MMP-7, and MMP-13 mRNA levels have been reported in Crohn’s disease biopsy specimens. MMP-2 and MMP-9 indicated increased protein secretion [155]. Jakubowska et al., [257] based on their research, also observed an increase in the concentration of MMP-2, MMP-7, and MMP-9. Researchers suggest that the characterized metalloproteinases may constitute a potential therapeutic target, and the use of their inhibitors may significantly reduce the progression of Crohn’s disease [133]. In studies in children, serum MMP-7 reflected disease activity [156,157]. Literature data indicate that the main biomarkers of inflammatory bowel diseases are four MMPs, i.e., MMP-3, MMP-7, MMP-9, and MMP-11.

## 5. Conclusions

The cause of IBD is multifactorial, with genetic as well as environmental, infectious, and immunological factors contributing to its development. Research has made significant progress in understanding the pathogenetic mechanisms of IBD. It is difficult to deny the widespread belief that IBD results from an extremely complex interaction between genetic and environmental elements, a dysregulated immune response, and changes in the microbiome, and that none of these factors alone can cause the disease. Moreover, the molecular response in IBD is dominated by T cells. In CD, Th1 and Th17 lymphocytes dominate, and interleukins produced by CD4 cells (mainly IL-17 and 22) and INF-γ are also important. In UC, Th2 lymphocytes predominate, resulting in the expansion of NK cells and the production of IL-13 and IL-5. It is worth noting that the MMP family also plays a key role in IBD patients. The primary biomarkers are MMP-1, MMP-2, MMP-7, MMP-8, MMP-9, MMP-12, MMP-13, MMP-14, and MMP-21. As shown in the review, their expression is associated with poor prognosis and increased inflammation. MMPs are mainly associated with inflammatory diseases. In colitis, the concentration of most MMPs is increased. Therefore, lowering the levels of MMPs may have a positive effect on preventing the development of inflammation. However, it should be noted that most MMPs have bidirectional effects, i.e., they participate in pathogenesis and promote the occurrence of malignant tumors, but also play a significant role in the progression of other healthy (non-cancerous) cells. Therefore, the multifaceted nature of MMPs as well as the multidirectionality of biological therapy requires further clinical trials to demonstrate which type of treatment is best for long-term follow-up.

## Figures and Tables

**Table 1:** UC disease severity index-prepared base on [1,2,3,4].

Descriptor (Score Most Severe Lesions)	Likert Scale Anchor Points	Definition
Vascular pattern	Normal (1)	Normal vascular pattern with arborization of capillaries clearly defined, or with blurring or patchy loss of capillary margins
	Patchy obliteration (2)	Patchy obliteration of vascular pattern
	Obliterated (3)	Complete obliteration of vascular pattern
Bleeding	None (1)	No visible blood
	Mucosal (2)	Some spots or streaks of coagulated blood on the surface of the mucosa ahead of the scope, which can be washed away
	Luminal mild (3)	Some free liquid blood in the lumen
	Luminal moderate or severe (4)	Frank blood in the lumen ahead of endoscope or visible oozing from mucosa after washing intraluminal blood, or visible oozing from a hemorrhagic mucosa
Erosions and ulcers	None (1)	Normal mucosa, no visible erosions or ulcers
	Erosions (2)	Tiny (≤5 mm) defects in the mucosa, of a white or yellow color with a flat edge
	Superficial ulcer (3)	Larger (>5 mm) defects in the mucosa, which are discrete fibrin-covered ulcers in comparison with erosions, but remain superficial
	Deep ulcer (4)	Deeper excavated defects in the mucosa, with a slightly raised edge

**Table 2:** Modified Truelove and Witts criteria-prepared base on [8,10].

Parameter	Mild	Moderate	Severe
Bloody stool per day, n	<4	4–6	>6
Pulse, beats per minute	<90	≤90	>90
Temperature, °C	<37.5	37.5–37.8	>37.8
Hemoglobin, g/dL	>11.5	11.5–10.5	<10.5
ESR, mm/h (or CRP, mg/L)	<20 (normal)	20–30 (<30)	>30 (>30)

**Table 3:** Types of tests that are used in the diagnosis of UC prepared based on [21,22,23,24].

Investigation	Type of Investigation	Common Findings in UC
Blood tests	Full blood count Urea and electrolytes C-reactive protein Vitamin D and bone profile Hematinic Liver biochemistry	Anemia, thrombocytosis, low vitamin D, and raised inflammatory markers
Histology	Recommend at least two biopsies from each bowel segment for histological assessment	No histological features are diagnostic of UC, but basal plasmacytosis, crypt atrophy/distortion, and villous surface irregularity are suggestive of UC
Stool cultures	*Clostridioides difficile* toxin assay MC&S	Should be negative if UC, but infections such as *C. difficile* can co-exist
Faecal calprotectin	Indicates migration of neutrophils to the lumen via the intestinal mucosa	A level of 50–100 μg/g has a high negative predictive value of 98–99% in the diagnosis of IBD
Endoscopy	In acute setting, flexible sigmoidoscopy Ileocolonoscopy is recommended in all patients to delineate disease extent, severity of inflammation and to exclude Crohn’s disease; also, for surveillance	Erythema, edema, loss of vascular pattern, blood, and ulcers/erosions
Imaging	Abdominal X-ray Thumbprinting, lead-piping, edema, and toxic megacolon Cross-sectional imaging: CT/MRI Bowel wall edema and inflammatory pseudopolyps	-

**Table 4:** Main genes involved in IBD pathogenesis [28,29].

Role/Pathway	Colitis Ulcerosa	Crohn’s Disease	IBD
Epithelial barrier	*GNA12*, *HNF4A*, *CDH1*, *ERRFI1*, and HLA allelic associations (mainly class II)	*MUC19*, *ITLN1*, *TCF4*, and *KCNN4*	-
Restitution	*ERRFI1*, *HNF4A*, *PLA2G2A/E*, and HLA allelic associations (mainly class II)	*STAT3*, *TCF4*, and *KCNN4*	*REL*, *PTGER4*, and *NKX2–3*
Solute transport	*AQP12A/B*, *SLC9A3*, *SLC26A3*, and HLA allelic associations (mainly class II)	*SLC9A4*, *SLC22A5*, *SLC22A4*, *TCF4,* and *KCNN4*	-
Paneth cells	-	*ITLN1*, *NOD2*, *ATG16L1*, *TCF4*, and *KCNN4*	*XBP1*
Innate mucosal defense	*SLC11A1*, *FCGR2a/B*, and HLA allelic associations (mainly class II)	*NOD2*, *ITLN1*, *TCF4*, and *KCNN4*	*CARD 9*, and *RER*
Immune cell recruitment	*IL8RA/IL8RB*, and HLA allelic associations (mainly class II)	*CCL11*, *CCL2*, *CCL7*, *CCL8*, *CCR6*, *TCF4*, and *KCNN4*	*MST1*
Antigen presentation	-	*ERAP2*, *LNPEP*, *DENND1B*, *TCF4*, and *KCNN4*	-
IL-23/Th17	*IL21*	*STAT3*	*IL23R*, *JAK2*, *TYK2*, *ICOSLG*, and *TNFSF15*
T-cell regulation	*IL2*, *TNFRSF9*, *PIM3, IL7R*, *TNFSF8*, *IFNG*, and *IL21*	*NDFIP1*, *TAGAP*, *IL2RA*, *TCF4*, and *KCNN4*	*TNFSF8*, *IL12B*, *IL23R*, *PRDM1,* and *ICOSLG*
B-cell regulation	*IL7R*, and *IRF5*	IL5, *IKZF1*, *BACH2*, *TCF4*, and *KCNN4*	-
Immune tolerance	*IL1R1/IL1R2*	IL27, *SBNO2*, and *NOD2*	IL10, and CREM
Autophagy	*PARK7*, and *DAP*	*ATG16L1*, IRGM, NOD2, *LRRK2*, *TCF4*, and *KCNN4*	*CUL2*
Apoptosis/ necroptosis	*DAP*	*FASLG*, and *THADA*	*PUS10*, and *MST1*
ER stress	*SERINC3*	*CPEB4*, *TCF4*, and *KCNN4*	*ORMDL3*, and *XBP1*
Carbohydrate metabolism	-	*GCKR*, *TCF4*, and *KCNN4*	*SLC2A4RG*
Intracellular logistics	*TTLL8*, *CEP72*, and *TPPP* HLA allelic associations (mainly class II)	*FGFR1OP*, and *VAMP3*	*KIF21B*
Oxidative stress	*HSPA6*, *DLD*, and *PARK7*	*PRDX5*, *BACH2*, *ADO*, *GPX4*, *GPX1*, *SLC22A4*, *LRRK2*, *NOD2*, *TCF4*, and *KCNN4*	*CARD9*, *UTS2*, and *PEX13*
Cell migration	*ARPC2*, *LSP1*, and *AAMP*	-	-

**Table 5:** Factors that increase the likelihood of developing IBD.

No.	Factor	CD	CU	IBD
1	smoking	+		
2	urban living	+		+
3	appendectomy	+		
4	tonsillectomy	+		
5	antibiotic exposure			+
6	oral contraceptive use			+
7	consumption of soft drinks		+	
8	vitamin D deficiency			+
9	non-Helicobacter pylori-like enterohepatic Helicobacter species			+

**Table 6:** Factors that reduce the likelihood of developing IBD.

No.	Factor	CD	CU	IBD
1	physical activity	+		
2	breastfeeding			+
3	bed sharing	+		
4	tea consumption		+	
5	high levels of folate			+
6	high levels of vitamin D	+		
7	H pylori infection	+	+	+

**Table 7:** Cytokines in inflammatory bowel disease (CD and UC) prepared based on [99,100,101,102,103,104,105,106,107,108,109].

Cytokines	Suggested Function	Appropriate Disease
OSM	Stem cell chemoattraction and tissue retention of neutrophils, monocytes, and T lymphocytes	CD/UC
CSF3	Increased tissue neutrophil survival	CD
IL1B	Costimulation in an inflammatory microenvironment	CD/UC
IL1A	Costimulation in an inflammatory microenvironment	CD/UC
IL6	Local and systemic inflammation, epithelial cell proliferation, and T cell activation	CD/UC
IŁ27	Th17 shift to inflammation via Th1	CD
IL11	Regulation of stem cells fibrosis	CD
CSF2	Neutrophil/monocyte stimulation	CD
IL22	Increases proliferation and production of antimicrobial peptides in the epithelium	CD
TNFSF13	Homeostasis and B cell differentiation	CD/UC
IL17A	Emergency granulopoiesis	CD/UC
TNF	Promotes acute phase proteins	CD/UC
IL12A	Differentiation Th1	CD
IL17F	Similar to IL17A but weaker	CD
TGFB2	Immune regulation, and inhibits proliferation	CD/UC
IL33	Alarming, tissue remodeling, cup cell proliferation, and Treg expansion	CD/UC
EBI3	Th17 shift to inflammation via Th1	CD/UC
TGFB3	Immune regulation, and inhibits proliferation	CD/UC
TGFB1	Immune regulation, and inhibits proliferation	CD/UC
LIF	Stem cell maintenance, and cell differentiation	CD/UC
CSF1	Monocyte stimulation	CD/UC
IL15	T-cell homeostasis	CD
IL32	Monocyte differentiation, and activation-induced cell death	CD
IL21	Th17 differentiation, and B cell homeostasis	CD
TSL	Activation of antigen-presenting cells	CD
IF	Activation of cellular immunity	CD/UC
TGFB3	Immune regulation, and inhibits proliferation	CD/UC
TL1A	Co-stimulation IFNG	UC
IL23A	Regulation of responses mediated by Th17 and IL-22	UC
IL16	Chemo attraction	UC
IL34	Growth and development of myeloid cells	UC
IL26	Antibacterial activity	CD/UC
IŁ29	Promotes epithelial antiviral functions	UC
IL2	Proliferation and survival of T cells	UC
IL37	Inhibits innate immunity	UC

**Table 8:** Expression and Function of Various Matrix Metalloproteinases in IBD.

MMP	Class	Expression in IBD Compared to Controls	Number of Controls	Number of Patients	Disease	Quantification Technique
MMP-1	Collagenases	Increased	8	8, 5, 6, and 7	U, UC, and CD	In situ hybridization
			Not stated	30	UC	Quantitative polymerase chain reaction (qPCR)
			Not stated	17, and 16	UC, and CD	Immuno-histochemistry
			Not applicable 20	Not applicable 30	Not applicable UC	Microarray Real-Time (RT-PCR)
MMP-2	Gelatinases	Unchanged	62	20, and 122	UC, and CD	ELISA
			14	23, and 22	UC, and CD	RT-PCR
MMP-3	Stromelysins	Increased	Not applicable	Not applicable	Not applicable	Microarray, in situ hybridization
			9	11	CD	In situ hybridization
			10	13, and 25	UC, and CD	ELISA
			16	23, and 24	UC, and CD	RT-PCR
			Not applicable	Not applicable	Not applicable	Microarray
MMP-7	Stromelysins	Increased	Not stated	Not stated	UC, and CD	Microarray, and RT-PCR
			Not stated	52	UC	Immuno-histochemistry
			Not stated	35	UC	RT-PCR
			4	25	UC	Immuno-histochemistry
			19	17, 23, and 19	UC, CD, and AP	qPCR
MMP-8	Collagenases	Increased	11	12, and 11	UC, and CD	Immuno-histochemistry
MMP-9	Gelatinases	Increased	Not applicable	Not applicable	Not applicable	Microarray, in situ hybridization
			9	11	CD	In situ hybridization
			9	31, and 13	UC, and CD	Zymography
			8	16	UC	qPCR
			Not stated	17, and 16	UC, and CD	Immuno-histochemistry
			Not applicable	Not applicable	Not applicable	Microarray
			Not applicable	Not applicable	Not applicable	Zymography
MMP-10	Stromelysins	Increased	21	21, and 22	UC, and CD	qPCR
			11	12, and 11	UC, and CD	Immuno-histochemistry
			Not stated	Not stated	UC, and CD	Microarray, and RT-PCR
			Not stated	5	IC	qPCR
MMP-11	Stromelysins	Unchanged	Not applicable	Not applicable	Not applicable	Microarray, in situ hybridization
MMP-12	Elastases	Increased	7	10, 7, and 14	UC, IC, and CD	In situ hybridization
			11	12, and 11	UC, and CD	Immuno-histochemistry
			Not applicable	Not applicable	UC, and CD	Microarray
MMP-13	Collagenases	Inconclusive	Not applicable	Not applicable	Not applicable	Microarray, in situ hybridization
			30	35, and 24	UC, and CD	Immuno-histochemistry
MMP-14	Membrane types	Inconclusive	Not applicable	Not applicable	Not applicable	Microarray, in situ hybridization
			14	23, and 22	UC, and CD	RT-PCR
MMP-17	Membrane types	Unchanged	Not applicable	Not applicable	Not applicable	Microarray, in situ hybridization
MMP-19	Other	Unchanged	Not applicable	Not applicable	Not applicable	Microarray, in situ hybridization
			5	24, 9, 7, and 20	UC, IC, CD	Immuno-histochemistry
MMP-23	Other	Increased	20	40, and 30	UC, and CD	RT-PCR
MMP-26	Other	Unchanged	5	24, 9, 7, and 20	UC, IC, CD	Immuno-histochemistry
MMP-28	Other	Decreased	Not stated	35	UC	RT-PCR
			5	24, 9, 7, and 20	UC, IC, CD	Immuno-histochemistry

**Table 9:** The pathogenetic role of cytokines in UC [209,210,211].

Cytokine	Mechanism	References
IL-9 (Cells Secreting Cytokine Th-9)	IL-9 (Th-9) IL-9 stimulation promoted claudin-2 expression while inhibited claudin-3 and occludin expression. Furthermore, SOCS3 overexpression rescued the IL-9-induced effects. Altogether, IL-9 participates in the pathogenesis of UC through STAT3/SOCS3 signaling pathway.	[209] Tian et al., 2018
IL-17 (Cells Secreting Cytokine Th17, and ILC3)	IL-17 promoted inflammatory cytokines (IL-1β, TNF-α) and chemokines responsible for leukocytes and neutrophils migration to inflamed tissues, in the absence of IL-23 supports the intestinal barrier through occludin regulation in tight junctions.	[210] Kałużna et al., 2022
IL-22 (Cells Secreting Cytokine Th17, and Th22)	IL-22 production is still an uncharted area, awaiting more detailed analysis on the transcription factors (TF) that help define their developmental pathways and phenotypic stability. L-22) has been extensively studied for its roles in maintaining mucosal barrier integrity, antimicrobial defense, cellular proliferation, and inflammation. The beneficial and pathogenic roles of IL-22 in various disease settings.	[211] Yan et al., 2021
